# Playing Super Mario 64 increases hippocampal grey matter in older adults

**DOI:** 10.1371/journal.pone.0187779

**Published:** 2017-12-06

**Authors:** Greg L. West, Benjamin Rich Zendel, Kyoko Konishi, Jessica Benady-Chorney, Veronique D. Bohbot, Isabelle Peretz, Sylvie Belleville

**Affiliations:** 1 Department of Psychology and Centre de Recherche en Neuropsychologie et Cognition, University of Montreal, Montreal, Quebec, Canada; 2 International Laboratory for Brain, Music and Sound Research (BRAMS), Montreal, Quebec, Canada; 3 Department of Psychology, University of Montreal and Institut universitaire de gériatrie de Montréal, QC, Canada; 4 Faculty of Medicine, Division of Community Health and Humanities, Memorial University of Newfoundland, Newfoundland, Canada; 5 Douglas Hospital Research Centre, Department of Psychiatry, McGill University, Verdun, Québec, Canada; Waseda University, JAPAN

## Abstract

Maintaining grey matter within the hippocampus is important for healthy cognition. Playing 3D-platform video games has previously been shown to promote grey matter in the hippocampus in younger adults. In the current study, we tested the impact of 3D-platform video game training (i.e., Super Mario 64) on grey matter in the hippocampus, cerebellum, and the dorsolateral prefrontal cortex (DLPFC) of older adults. Older adults who were 55 to 75 years of age were randomized into three groups. The video game experimental group (VID; *n* = 8) engaged in a 3D-platform video game training over a period of 6 months. Additionally, an active control group took a series of self-directed, computerized music (piano) lessons (MUS; *n* = 12), while a no-contact control group did not engage in any intervention (CON; *n* = 13). After training, a within-subject increase in grey matter within the hippocampus was significant only in the VID training group, replicating results observed in younger adults. Active control MUS training did, however, lead to a within-subject increase in the DLPFC, while both the VID and MUS training produced growth in the cerebellum. In contrast, the CON group displayed significant grey matter loss in the hippocampus, cerebellum and the DLPFC.

## 1. Introduction

Lower grey matter in the hippocampus is a significant biomarker for numerous neurological and psychiatric disorders across people’s lifespan including disorders that specifically impact older adults such as Mild Cognitive Impairment and Alzheimer’s disease [1–3]. The impact of learning on the hippocampal system is only beginning to be better understood. The neuroplastic nature of the hippocampus was first reported in humans by researchers who examined London Taxi drivers who underwent rigorous spatial memory training to learn the city’s layout and routes (commonly referred to as “the knowledge”). It was shown that London Taxi drivers displayed more grey matter in the posterior hippocampus compared to a matched control group [4]. In a subsequent study, this relationship was shown to be causal by using a longitudinal design that followed a sample of potential London taxi drivers who underwent the multi-year training program to acquire “the knowledge”. It was found that people who successfully completed the training program displayed increased grey matter in the hippocampus at post-training while people who were not successful, displayed no significant increase within this neural structure [5]. It is hypothesized that the type of learning that successful trainees underwent involves building relationships between environmental landmarks to create a detailed internal representation of the environment or a *cognitive map* [6], a process that relies centrally on the hippocampus [7–12]. For example, learning the relationship between landmarks (e.g., buildings, trees, boulders, rivers etc.) allows for a flexible use of this information to navigate to a destination point that is independent of the position of the observer. Further, post-training grey matter changes positively correlate with functional neural activity recorded during a training task’s execution [13]. This confirms that observed increases in grey matter is linked to task-specific neural processing.

This evidence has encouraged research into using cognitive training techniques that would target the hippocampal system with the aim to prevent cognitive decline associated with decreased hippocampal grey matter [1, 2]. One technique that has shown promise in promoting targeted hippocampal growth in young adults has been with the use of 3D-platform video game training. Findings from a correlational analysis found that time spent playing platform video games, such as Super Mario 64 and puzzle/logic games such as Tetris, was associated with more grey matter in the entorhinal cortex [14], a structure that is both structurally and functionally connected to the hippocampus. A follow-up longitudinal training study conducted by the same research team showed that young adults who trained on Super Mario 64 for two months displayed a significantly different amount of grey matter in the hippocampus when contrasted with a passive no-contact control group [15]. Corroborating evidence supporting the positive impact of 3D-platform video game training on memory which was previously shown to be dependent on the hippocampus, in young adults, was obtained using a longitudinal training design with behavioral measures. It was demonstrated that young adults who trained on Super Mario 3D world showed increased spatial and episodic memory performance compared to people who trained on a 2D-control game [16]. Together, these results suggest that 3D-platform video game training can be beneficial to hippocampus dependent grey matter and memory.

The relationship between the hippocampus and 3D-platform games is thought to be driven by the fact that 3D-platform games require the use of spatial memory processes to build a cognitive map of in-game environments and therefore requires learning that depends on the hippocampus [6, 7, 9, 12]. Because of this, 3D-platform video game training holds promise to be applied to populations that are at increased risk for developing neurodegenerative disorders associated with decreased hippocampal integrity, such as older adults. No study to date has shown that video game training can directly increase grey matter in the hippocampus of older adults. The aim of the present study was therefore to directly test if 3D-platform video game training can increase grey matter in neural structures know to become dysfunctional during ageing.

Specifically, we hypothesized that older adults who trained on the 3D-platform game Super Mario 64 for 6 months would benefit from its prominent spatial navigation components [15, 17]. We therefore predicted that older adults who trained on platform games such as Super Mario 64 would display increased grey matter in the hippocampus.

Furthermore, because of Super Mario 64’s requirement for fine motor coordination, we also expected 3D-platform training to increase grey matter in the cerebellum. Indeed, both Kühn et al., 2014 [15] and West et al., 2017 [18] observed increased grey matter in the cerebellum of younger adults after Super Mario 64 training. In addition to the cerebellum’s involvement in motor control, this structure has been linked to short-term memory, procedural learning and episodic memory performance [19]. As a result, the learning required of participants in the virtual environment, which is both procedural and spatial in nature, was predicted to increase grey matter in the cerebellum.

Finally, Super Mario 64 requires ample planning and the internal storage and manipulation of in-game information. We therefore predicted that training would increase grey matter in the dorsolateral prefrontal cortex (DLPFC) [15]. We also predicted that Super Mario 64 training would have a positive effect on cognitive performance in older adults, namely in short-term memory performance and in an estimate of overall cognitive health as measured by the Montreal Cognitive Assessment (MoCA).

To isolate the specific impact of video game training (VID group) on cognitive performance and grey matter within our defined regions of interest, two control groups were included in the study’s design: an active music control group who trained on the piano for 6 months (MUS group) and a passive no-contact control group (CON group). Previous evidence has clearly demonstrated that different training demands will differentially impact neural networks and structures [20]. We therefore predicted that music training would have little impact on the hippocampal system due to its limited spatial memory training component; however, cognitive function associated with planning and reasoning should be involved in music training, resulting in increased DLPFC grey matter. The CON group was expected to not show any increase in grey matter in any of the identified regions of interest and display no notable change in cognitive performance at the end of the 6 month training period.

## 2. Materials and methods

### 2.1 Participants & Randomization procedure

Participants were recruited into the study from the CRIUGM participant pool. The study received ethical approval from the Comite conjoint d’evaluation scientifique–Regroupment Neuroimagerie/Quebec (CES-RNQ). Participants gave informed consent in writing. Participants were pre-screened to ensure that they did not have any present or past major illness, were not taking any psychiatric medications or medication known to have an impact on cognition, they were MRI compatible and that they were a non-video game player and a non-musician. Participants also were screened for MCI using the Montreal Cognitive Assessment (MoCA) (all participants scored ≤ 25 [21]). To be considered a non-video game player, participants had little to no experience with commercial video games (e.g., games played on a computer or game console) during their lifetime. Casual games such as computerized card or puzzle games were not considered to be video games.

All participants were randomized into one of three groups. Randomization was done by an independent research assistant, using a predefined randomization table prior to contacting participants to ensure that they were blind to the existence of the other two conditions. Randomization was stratified using a covariate-adaptive randomization. Each factor was stratified into two categories. For the factor of age there were “younger” (55–64 yrs) and “older” (65–75 yrs); for the factor of education there was low (< 16 yrs) and high (> 16 yrs); and for the factor of gender there was female and male. Because participants were recruited from a database, age, education level, and gender of each participant were known before they were contacted and it was thus possible to stratify randomization on the basis of these three factors.

To reduce the impact of expectancy on test-retest effects, all participants were told that they were expected to improve in performance. Participants in the VID group were told that there was evidence that video game training enhances cognitive abilities, and that video game training in older adults was expected to improve those abilities. Participants in the MUS group were told that there was evidence that musicians have enhanced cognitive abilities, and that we expected musical training to improve those abilities. Finally, the CON group was told that we were investigating test-retest effects, and that they were expected to improve on all tasks. All participants were debriefed about the other groups at the end of the final testing session.

Fourty-eight participants in total were recruited into the study. Using the stratified randomization procedure, 15 participants were assigned to the VID group, 14 participants were assigned to the MUS group and 15 participants were assigned to the CON group. During the study, 2 participants withdrew from the MUS group, 2 withdrew from the control group, while 11 withdrew from the VID group. To account for the higher attrition rate within the VID group, an additional four participants were assigned who were matched for the age, gender and education of the other two groups, however, the stratified randomization procedure was not used. This resulted in a total of 8 participants completing the training within the VID group. The demographics of the participants within each group are presented in Table 1.

**Table 1:** Demographic information for each experimental group.

	Age (+/- S.D.)	Education (+/-S.D.)	Gender (% of females)
**VID Group**	69.3 (5.7)	15.2 (3.2)	50%
**MUS Group**	67.7 (4.3)	14.7 (2.3)	83.3%
**CON Group**	66.9 (3.9)	17.5 (2.3)	76.9%

### 2.2 Training procedure

Video game and music training lasted 6 months. In all cases, participants kept a record of their daily training progress and were asked to complete a minimum of 30 minutes of training at least five days a week, although some completed more than this amount.

**Video game training:** Video game training was done at home using the Nintendo Wii console system equipped with a Wii Classic Controller. All participants in this group trained on Super Mario 64. Two participants completed all task within Super Mario 64 before the completion of the 6 month training period. In these cases, they continued to train on a very similar game, Super Mario Galaxy, until the end of the training period. Super Mario 64 and Super Mario Galaxy are three-dimensional platform games where the player is tasked with exploring a virtual environment to search for stars (tokens). When enough stars are collected through completing in-game goals, the player can then progress further into the game and will encounter new environments to explore.

After the participant completed the pre-tests, a research assistant installed the Nintendo Wii to the participant’s home television. The research assistant then gave an initial orientation to the participant to teach them how to turn on the Nintendo Wii and access the Super Mario 64 game. This was followed by a custom in-game orientation which taught the participant to move the character around the virtual environment. At this point, some participants encountered certain challenges associated with maneuvering the character. Some had issues with understanding the game’s mechanics or spatial memory or motor coordination. Further, Super Mario 64 was not designed to be played by someone with little or no video game or computer experience and has a very steep learning curve. For this reason, the research assistant returned to the participant’s home for up to three additional supervised 2 hour training sessions to teach the participant how to properly maneuver the character and progress through the game. After this, participants were given a custom made instruction booklet which outlined how and where to collect all the stars for the first four levels. This allowed participants to learn the game’s mechanics in further detail and practice the basic motor coordination that was required. After this point, participants had to search for and obtain the stars within each remaining level without any assistance from the research team.

**Music training (Active control):** Piano training was done at home using Synthesia software, and an 88-key M-Audio MIDI piano. First, the research assistant installed and calibrated the piano to work on the participant’s home computer. Next, they completed an introductory lesson that included introductory information about music, detailed instructions on how to use Synthesia, and directions on how to record their progress. Introductory music information included lessons about note names, how to place hands on the piano, and how to synchronize performance with the information on the screen and the metronome. A set of introductory lessons, and beginner piano music was installed on the computer. Participants were told to start with the lessons, and once they were comfortable with the lessons, to try out some of the introductory songs. Participants were encouraged to move at their own pace, but to try to master a given lesson or song before moving on. Sometimes participants would work on a lesson and song simultaneously.

**Passive control group:** The passive control group had no contact with the research team during the 6 month period other than to complete the pre- and post-testing sessions.

### 2.3 Outcome measures

All participants underwent a pre- and post-training testing session which included cognitive tests and a structural MRI scan.

**The Montreal cognitive assessment:** The Montreal Cognitive Assessment (MoCA) [21] is commonly administered to assess general cognitive function. Lower scores on the MoCA indicate lower cognition and a greater risk for developing Mild Cognitive Impairment (MCI) and Alzheimer’s disease.

**Short-term memory performance:** A short-term memory test using speech sounds [22] was administered at pre- and post-training. Two non-sense monosyllabic syllables (‘‘ran-bij”) were selected to create the verbal material. The syllables were chosen so that they did not share letters and they did not form real words in isolation or when combined. The sequences were binary, meaning that they were composed of the two items repeated in random order (eg. ran–bij–bij–ran). A male voice was used to record the syllables which were read at a rhythm of one item per second. These two speech sounds were randomly combined into 28 sequences of increasing length starting at two syllables and going up to eight syllables. For each sequence participants were asked to listen to and memorize the sequence. After the sequence finished there was a 1 second delay, and a second sequence was played. The test sequence was identical to the learned sequence for half the trials, and on the other half of the trials the sequence differed by a single syllable. Participants were asked to determine if the test sequence was the same or different than the learned sequence. Sequences were presented with the shortest one first, and the length increased as the experiment progressed. Four different trials were presented at each sequence length. A higher score represents a higher level of short-term memory performance.

#### Voxel-based morphometry

Participants were scanned on a Siemens TIM Trio 3T MRI system (Siemens Medical Solutions, Erlangen, Germany), using the Siemens 12-channel receive-only head coil at L'Unité de Neuroimagerie Fonctionnelle (UNF) of the Centre de recherche de l'Institut universitaire de gériatrie de Montréal. An MPRAGE anatomical scan of approximately nine minutes was performed. A three-dimensional gradient echo acquisition was used to collect 160 contiguous 1 mm T1-weighted images in the sagittal plane (TR = 2300 ms, TE = 2.91 ms, flip angle = 9°, FOV = 256 mm^2^, voxel size = 1 mm x 1 mm x 1 mm resolution).

Changes in grey matter were measured using voxel-based morphometry (VBM). VBM is a computational approach to neuroanatomy that measures differences in the local density of brain tissue through a voxel-wise comparison of multiple brain images [23]. MRI images were run through a bioinformatics pipeline (bpipe). The images were first corrected for intensity non-uniformity (shading artifacts) using the N4 software package [24] and then spatially normalized by linear transformation using ICBM 152 atlases [25]. The neck was then removed from the scans using a head mask of the brain with open-source MINC tools (http://www.bic.mni.mcgill.ca/ServicesSoftware/MINC). The BEAST algorithm was used to linearly normalize the intensity of scans, masked individually using a brain mask generated in model space [26]. INSECT (Intensity Normalized Stereotaxic Environment for the Classification of Tissues) [27] was used to automatically label voxels as white matter, grey matter, cerebrospinal fluid, or background. White matter, grey matter, and cerebrospinal fluid were extracted from the brain and blurred using a 4mm FWHM (full-width at half-max) Gaussian kernel. Analyses were run using RMINC (http://launchpad.net/rminc), which operates using the R statistical package (http://www.r-project.org).

We chose our regions of interest (ROIs) based on our a priori hypotheses was set[7, 9, 10], namely the hippocampus, DLPFC and the cerebellum [15]. Regions of interest (ROIs) were structurally defined in advance of data collection, based on our a priori hypotheses. Because of this, an uncorrected threshold of p < 0.001 for the peak voxel within ROIs was set. We also report effects that pass small volume correction, which is based on an even more conservative statistical threshold. In these cases, the resultant p-value accounted for the number of voxels in the hippocampus [4, 8]. In both cases, the voxels within our ROIs needed to pass a more conservative threshold compared to voxels outside these regions.

As recommended by Lövdén et al., 2013[28], we first conducted group (VID; MUS; CON) by time (pre-training; post-training) interaction analyses. Interaction analyses were corrected for multiple comparisons using the small volume correction (SVC) procedure with a significance threshold of *p* < 0.05. To qualify observed interaction effects that pass small volume correction, we conducted planned ROI analyses using paired sample *t*-test within each training group to investigate changes in grey matter at an uncorrected threshold of *p* < 0.0001.

#### Note

There were a number of additional measurements taken to examine the impact of music training on auditory cognition. The purpose of this paper is to report the benefits of video-game playing on tasks where video-game training was hypothesized to have a positive impact on the brain. Results related to the benefits of music training on audition will be reported elsewhere.

## 3. Results

### 3.1 Equivalency of groups at pre-test

Because of the higher attrition rate in the VID group, there was increased concern regarding the equivalency of groups at pre-test. We therefore tested if there were any group differences in grey matter at pre-test within our identified ROIs and found no differences even at a more liberal uncorrected threshold of *p* < 0.05. This confirmed that there were no significant group differences at pre-test in any ROI. Group equivalency was further confirmed when examining pre-test MoCA scores using a one-way ANOVA (CON: 26.61; MUS: 28.16; VID 26.93; *F*(2,30) = 2.18, *p* > 0.1). Planned contrasts also revealed no significant differences (*t*s <1).

### 3.2 Training performance

On average, participants in the VID group trained for an average of 72 h (S.D. = 11.3) and participants in the MUS group trained for an average of 83 h (S.D. = 34.3).

### 3.3 Cognitive tests

When examining the MoCA scores, planned pairwise *t*-tests were used to contrast the MoCA scores before and after training within each group. A one-tailed test was employed due to a priori direction of our hypothesis. This revealed a significant increase at post training in the VID group (*t*(7) = 2.1, *p* <0.05; See Table 2 & S1 Fig), while the MUS and CON group did not display a significant increase (*t* s< 1). Planned pairwise *t*-tests were again used to examine data from the short-term memory task. This revealed a significant increase in the VID group at post-training (*t*(7) = 3.3, *p* <0.05; See S1 Fig) while no significant difference was observed in the MUS or CON group (*t*s < 1).

**Table 2:** Summary of behavioural results.

	Pre-training	Post-training	*t* value	*p* value
**CON Group**				
*MoCA*	26.6 ± 2.14	27.1 ± 1.85	0.98	*p* = 0.35
*Short-term memory*	23.7 ± 2.9	23.2 ± 3.3	0.58	*p* = 0.58
**MUS Group**				
*MoCA*	28.2 ± 1.9	28.2 ± 2.1	0.19	*p* = 0.85
*Short-term memory*	24.3 ± 2.7	24.8 ± 1.8	1.0	*p* = 0.32
**VID Group**				
*MoCA*	26.9 ± 1.3	28.3 ± 1.4	2.1	*p* < 0.05 (one-tailed)
*Short-term memory*	23.0 ± 2.6	25.0 ± 2.9	3.3	*p* < 0.05

### 3.4 Voxel-based morphometry

**Fig 1:**
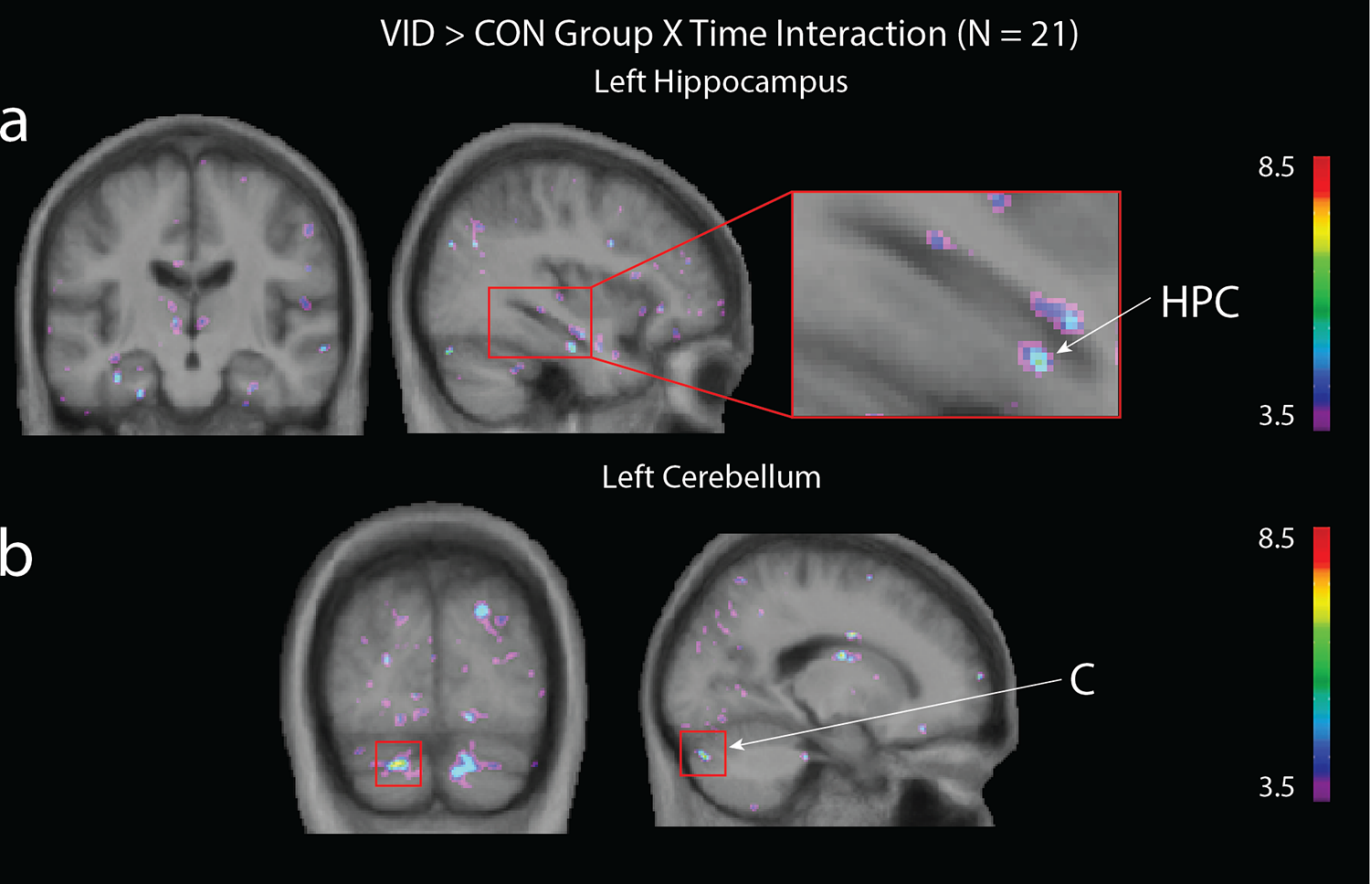
Group (VID; CON) x Time (pre-test; post-test) interaction. A significant effect was observed in the **(a)** left hippocampus (HPC; (x = -36, y = -16, z = -24; t = 5.69; p < 0.05, corrected) and **(b)** left cerebellum (C; cerebellum (x = -19, y = -83, z = -33 t = 7.57; p < 0.05 corrected).

**Fig 2:**
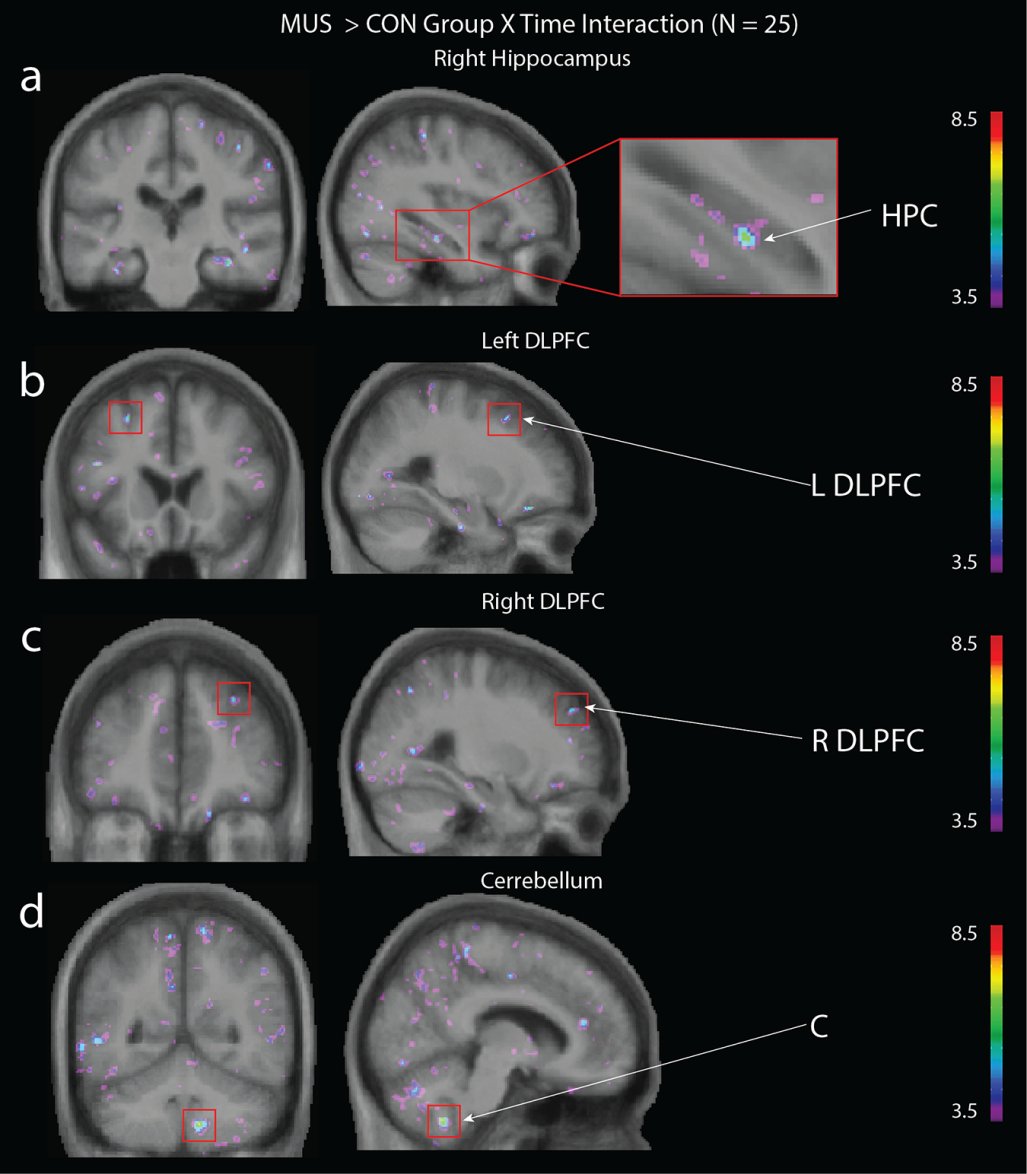
Group (MUS; CON) x Time (pre-test; post-test) interaction. A significant effect was observed in the **(a)** right hippocampus (HPC; (x = 35, y = -24, z = -17; t = 6.21; p < 0.05, corrected), **(b & c)** left DLPFC (x = -26, y = 19, z = 49; t = 5.79; p < 0.05, corrected) and right DLPFC (x = 27, y = 45, z = 35; t = 5.67; p < 0.05, corrected) and **(d)** the right cerebellum (C; x = 9, y = -55, z = -48; t = 6.79; p < 0.05, corrected).

We next tested for changes in grey matter within the three identified regions of interests: the hippocampus, cerebellum, and DLFPC (see Table 3). We conducted three Time x Group analyses to contrast the VID and CON group, the MUS and CON group and the VID and MUS group. Results of the Time X VID/CON group analysis (Fig 1) revealed a significant interaction in the left hippocampus (x = -36, y = -16, z = -24; *t* = 5.69; *p* < 0.05 *SVC*) and in the left cerebellum (x = -19, y = -83, z = -33 *t* = 7.57; *p* < 0.05 *SVC*). The Time X MUS/CON group analysis (Fig 2) revealed a significant interaction in the right hippocampus (x = 35, y = -24, z = -17; *t* = 6.21; *p* < 0.05, *SVC*), left DLPFC (x = -26, y = 19, z = 49; *t* = 5.79; *p* < 0.05, *SVC*), right DLPFC (x = 27, y = 45, z = 35; *t* = 5.67; *p* < 0.05, *SVC*) and right cerebellum (x = 9, y = -55, z = -48; *t* = 6.79; *p* < 0.05, *SVC*). The VID/MUS group analysis did not reveal any significant interaction that passed small volume correction.

**Table 3:** Summary of VBM results.

	Area	Peak coordinates (MNI)	*t* value
**VID / CON Interaction**	left hippocampus	x = -36, y = -16, z = -24	5.69 SVC
	left cerebellum	x = -19, y = -83, z = -33	7.57 SVC
**MUS / CON Interaction**	right hippocampus	x = 35, y = -24, z = -17	6.21 SVC
	left DLPFC	x = -26, y = 19, z = 49	5.79 SVC
	right DLPFC	x = 27, y = 45, z = 35	5.67 SVC
	right cerebellum	x = 9, y = -55, z = -48	6.79 SVC
**VID Pre-post contrast**	left hippocampus	x = -33, y = -13, z = -20	7.09
	right hippocampus	x = 32, y = -29, z = -12	6.19
	left cerebellum	x = -40, y = -67, z = -51	9.38
**MUS Pre-post contrast**	right DLPFC	x = 49, y = 29, z = 6	8.11
	right cerebellum	x = 14, y = -68, z = -59	7.08
**CON Pre-post contrast**	left hippocampus	x = -29, y = -18, z = -24	*-6*.*34*
	right hippocampus	x = 31, y = -7.9, z = -27	-6.25
	left cerebellum	x = -5, y = -64, z = -26	-8.81
	right cerebellum	x = 4, y = -67, z = -26	-8.10
	right DLPFC	x = 34, y = 39, z = 9	-5.37

**MNI** = Montreal Neurological Institute; **SVC** = *t* value passes small volume correction

**Fig 3:**
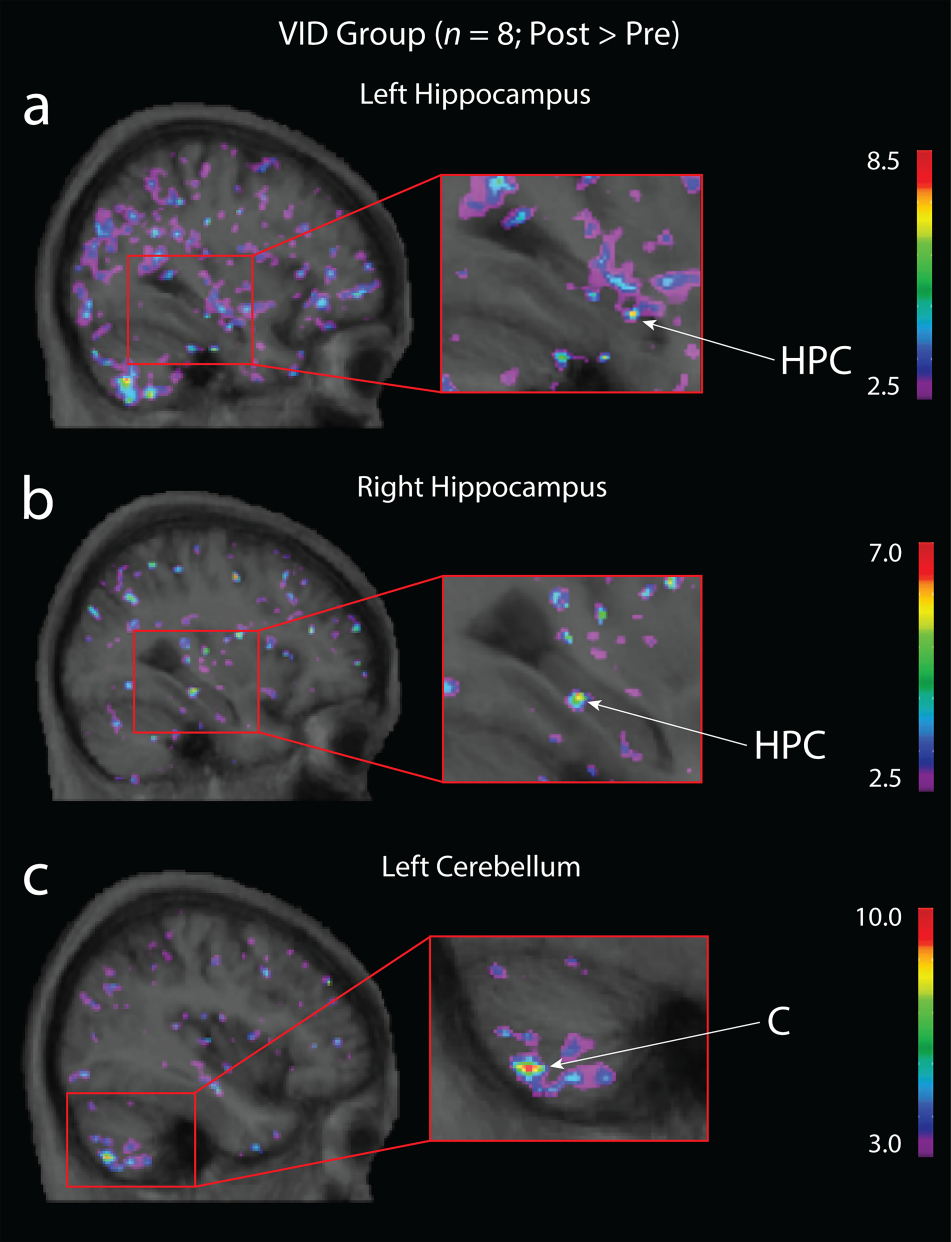
Planned within-subject contrast for the VID group. Increased grey matter in the **(a)** left hippocampus (x = -33, y = -13, z = -20; t = 7.09, p < 0.0001), **(b)** right hippocampus (x = 32, y = -29, z = -12; t = 6.19, p < 0.0005) and **(c)** left cerebellum (x = -40, y = -67, z = -51; t = 9.38, p < 0.00005) after older adults completed 6 months of video game training. Significant peaks in hippocampus = HPC; significant peak in cerebellum = C.

**Fig 4:**
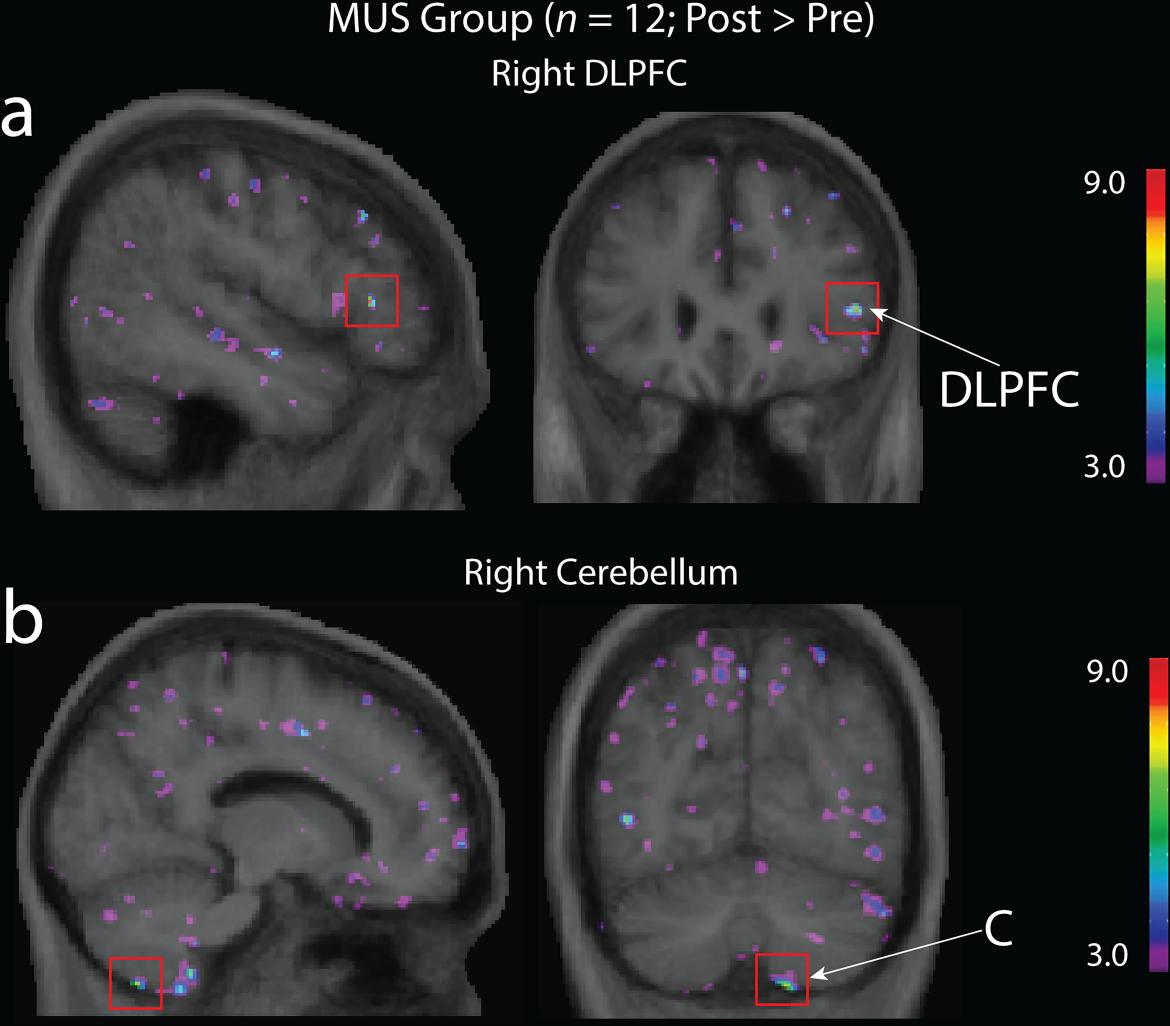
Planned within-subject contrast for the MUS group. Increased grey matter in **(a)** the right DLPFC (x = 49, y = 29, z = 6; t = 8.11, p < 0.000005) and **(b)** cerebellum (x = 14, y = -68, z = -59; t = 7.08; p < 0.0001) after older adults completed 6 months of music training. Significant peak in dorsolateral prefrontal cortex = DLPFC; significant peak in cerebellum = C.

To further qualify the observed interaction effects and investigate if growth or atrophy within a single group were driving these effects, we conducted planned ROI analyses using paired sample *t*-test within each training group at an uncorrected threshold of *p* < 0.0001. In the VID group, a significant increase in grey matter was observed bilaterally in the hippocampus (left: x = -33, y = -13, z = -20; *t* = 7.09, *p* < 0.0001; right: (x = 32, y = -29, z = -12; *t* = 6.19, *p* < 0.0005; Fig 3) and in the left cerebellum (x = -40, y = -67, z = -51; *t* = 9.38, *p* < 0.00005; Fig 3). In the MUS group, a significant increase in grey matter was observed in the right DLPFC (x = 49, y = 29, z = 6; *t* = 8.11, *p* < 0.000005) and the right cerebellum (x = 14, y = -68, z = -59; t = 7.08; p < 0.0001; Fig 4). No other significant increases were observed in any other region of interest or group. We then examined if any of the experimental groups experienced decreased grey matter over the study’s 6 month period. This revealed a significant decrease in grey matter in the passive control group within the hippocampus bilaterally (left: x = -29, y = -18, z = -24; *t* = -6.34, *p* < 0.00005; right: x = 31, y = -7.9, z = -27; *t* = -6.25, *p* < 0.00005), the cerebellum bilaterally (left: x = -5, y = -64, z = -26; *t* = -8.81, *p* < 0.00005; right: x = 4, y = -67, z = -26; *t* = -8.10, *p* < 0.00005) and the right DLPFC (x = 34, y = 39, z = 9; *t* = -5.37, *p* < 0.0001; See S2 Fig). No significant atrophy was observed in any of the ROIs in either the MUS or VID training groups, even at a more liberal threshold of *p* < 0.05.

Together, these results support the hypothesis that VID training specifically resulted in increased hippocampal grey matter, while MUS training appeared to have preserved tissue in this structure, but no within-subject growth was observed. MUS training, however, directly contributed to growth in the DLPFC and both VID and MUS groups displayed increased grey matter in the cerebellum after training.

No other significant results were found when performing whole brain comparisons.

## 4. Discussion

We investigated the impact of 3D-platform video game training on hippocampal grey matter and memory performance in older adults. Analyses revealed a significant Group x Time interaction within the hippocampus in both the VID and MUS groups when each contrasted with the CON group. Planned comparisons revealed that these interactions were driven by both post-training growth in the VID group and post-training atrophy in the CON group. No direct within-subject growth in the hippocampus was observed in the MUS group, thereby suggesting that the significant MUS/CON Group x Time interaction observed in the hippocampus was driven by the observed reduction in grey matter within the CON group.

Interaction effects were also observed in the DLPFC and cerebellum. Participants in both the VID and MUS group displayed a significant interaction in the cerebellum when contrasted with the CON group and both the VID and MUS group displayed a direct within-subject increase in this structure when planned comparisons were conducted. The MUS group uniquely displayed a Group x Time interaction in the DLPFC when contrasted with the CON group and planned within-subject comparisons confirmed that this was at least partly driven by growth in the MUS group.

Participants in the CON group uniquely displayed reduced grey matter in the hippocampus, cerebellum, and the DLPFC at the end of the 6 month period. This observation replicated results reported by Lövdén et al., 2012 [29] who observed that healthy older adults in a passive control group of a longitudinal study displayed atrophy in the hippocampus over a period of four months. Importantly, participants in the VID and MUS group showed no significant decrease in grey matter in any of the identified regions of interest, even at a more liberal uncorrected threshold of *p* < 0.05. Together, our current results provide support for the hypothesis that new cognitive activities can prevent the negative effect of age on hippocampal grey matter, and that casual video game training can directly increase grey matter in the hippocampus and improve memory abilities.

Training had a differential impact on certain measures of cognitive performance. The VID group uniquely showed an increase in short-term memory performance and the MoCA at post-training. Further, increases in short-term memory performance observed correlated with change in hippocampal grey matter across all participants. However, it is likely that this correlation was driven in part by significant grey matter loss in the control group (See S3 Fig) More research is needed to directly link gains in short term memory performance with increased hippocampal grey matter integrity after video game training.

How does 3D-platform training promote increased hippocampal grey matter in older adults? We hypothesize that the design of 3D-platform video games bias people towards hippocampus-dependent spatial learning. This is achieved through the building of relationships between landmarks in the virtual environment to create a cognitive map [6, 17]. For example, learning the relationships between landmarks (e.g., buildings, trees, rivers etc.) results in the flexible use of environmental information to navigate to a given destination point from any position in the environment. The encoding and retrieval of spatial memory from an internal cognitive map relies centrally on the hippocampus [30–33]. This direct relationship between hippocampus-dependent learning that relies on the use of external landmarks and grey matter in the hippocampus has been shown to be causal. For example, in a rodent study, mice were trained to find a platform through using either the surrounding landmarks in the testing room (hippocampus-dependent training) or a single ‘beacon’ stimulus which directly identified the platform’s location (non-hippocampus-dependent training). It was found that mice in the hippocampus-dependent training showed increased grey matter in the hippocampus after training while mice in the non-hippocampus-dependent training group instead showed increased grey matter in the striatum [12]. In humans, it was found that right hippocampal grey matter predicted ability to identify the relative locations of buildings on a university campus while blindfolded (i.e., relying on an internal cognitive map) [34]. Further, navigation performance using landmarks was positively correlated with hippocampal grey matter in older adults [35]. Related to this, both young and older adults who use hippocampus-dependent navigation strategies show greater fMRI activity [9, 11] and grey matter [7, 10] in the hippocampus. Therefore, increased hippocampus-dependent learning during 3D-platform training is likely the cause of the observed increased grey matter in the hippocampus.

Video game training and music training was also shown to increase grey matter in the cerebellum in older adults. The cerebellum is involved in balance and motor learning and control [36]. Further, grey matter loss in the cerebellum is related to decreased sensorimotor performance [37] and balance [38] in older adults. Our results therefore suggest that learning and efficiently executing new motor commands during 3D-platform video game training and music training has the potential to improve balance controlled by the cerebellum. Future research should examine the direct impact of 3D-platform video game and music training on measures of balance and gait in older adults to further explore this relationship.

In addition to the grey matter effects observed in the VID group at post-training, the MUS group uniquely displayed significant growth in the DLPFC. The DLPFC is involved in executive function, cognitive flexibility, planning, and inhibition, which are all functions that music training is thought to enhance [39]. We did not observe a significant increase in the DLPFC in the VID group, which was unexpected. It is possible that 3D-platform training does positively impact frontal structures of older adults, however, this effect could be weaker than the observed impact on the hippocampus and/or could require a larger training exposure. Further, no atrophy was observed in the DLPFC after 3D-platform training, whereas the CON group did display atrophy in this region. These findings suggest that 3D-platform training likely engaged the DLPFC more so than the passive control condition. Related to this, our current results also suggest that in addition to promoting grey matter growth, learning to play a new instrument or a 3D-platform video game may counteract age-related atrophy in the hippocampus, cerebellum, and DLPFC. Only the CON group showed significant grey matter decreases in the hippocampus, cerebellum, and DLPFC. In contrast to the passive CON group, both training groups did not display any significant reduction in grey matter in any of our defined regions of interest. In other words, learning new tasks could be related to preserving brain tissue within our identified ROIs in addition to targeted, task-dependent growth specific to the training task.

How can music and video game training cause specific changes in neural structures? With respect to changes in grey matter, as measured by VBM, it has been established that cognitive training can target specific neural structures that support the performance of the cognitive task in question. This can include effects based on both sensorimotor recruitment and the use of cognitive processes such as spatial or working memory. It, however, can be difficult to pinpoint exactly which aspect of training produces specific changes in neural structures. This underscores the need for future follow up research to establish which specific aspects of music and video game training caused the observed effects in the current paper. Another aspect to consider is the fact that structural changes observed by way of VBM are not necessarily caused by one sole process. A recent review by Zatorre, Fields & Johansen-Berg (2012) [40] explains that observed changes in grey and white matter likely involves many interconnected structural changes involving various cell types. More specifically changes in gray matter could indicate a change in neurogenesis, synaptogenesis or neuronal morphology. Specifically linking one of these processes to observed changes in grey matter using VBM based on T1-weighted images is, however, difficult to establish in human samples. Nonetheless, mouse work using both MRI and histological techniques has established that training related grey matter changes involving the hippocampus were directly linked to axonal growth cones [12]. Future research is needed to establish this relationship in humans. Another factor could be related to molecular changes as a result of training. For example, it has been found that exercise’s impact of hippocampal grey matter is mediated by an increase in Brain Derived Neurotropic Factor (BDNF) [41]. Further, it is also know that the BDNF valine (val) to methionine (met) polymorphism at codon 66 (val66met) is associated with less efficient secretion of BDNF leading to smaller hippocampal volumes [42] and dysfunction of the hippocampus [43]. Conversely, the val homozygous genotype is associated with increased hippocampal volumes [42]. It is possible that cognitive training could have an impact on BDNF, which interacts with gene polymorphism to produce changes in grey matter. These factors should be measured in future research.

The 3D-platform video game training produced positive grey matter increases in brain regions known to decline with age and are related to aging-related cognitive decline. The final sample size was, however, smaller in the VID training group. This was primarily associated with the steep learning curve within the design of Super Mario 64 and issues with learning the proper motor coordination needed to progress successfully through the game independently. Given this fact, we have decided to include a discussion about what can be learned from the attrition in the video-game group.

One the one hand, the final sample size in the VID group is lower; however, the attrition from the video game group provides some interesting insights into the usefulness of using video-games as a form of cognitive rehabilitation. In the current study, the active control group learned to play piano through a video-game interface, and attrition in this group was comparable to the control group. At the same time, learning a musical instrument has long been considered a useful skill, and worthwhile activity. The comparable rates of attrition in the music and control group suggest that the current cohort of older adults have positive associations with piano playing. The greater attrition in the video-game group suggests that this cohort has more negative associations with video-game playing. However, it can be argued that people who completed the video-game training had similar levels of motivation compared to people who completed the music training. Further, recent research has discovered many positive associations with video game playing. These benefits include enhanced cognitive abilities in younger adults [14, 15, 44–46] and as we demonstrate here, in older adults as well. Thus, as the current cohort of middle-aged and young adults ages, future cohorts of older adults will likely be more comfortable with using video-games as a form of cognitive intervention. This would reduce attrition rates because future cohorts of older adults would be motivated to complete the video-game training replicating the current video-game and music–training groups.

Along these lines, the current sample provides an insight into the specific effectiveness of video-game training that may not be testable in the next generation. Recruiting non-video game players over the age of 55 was relatively easy in the current study, while finding non-musicians was much harder. In fact, most participants in this study had completed some music education in primary school, while none of the participants had any experience with video-games. In the future it will be harder to find non video game players. Market research from the ESA claims that from 2014–2016 the average age of a video game player increased from 31–35 years of age. In the same research the ESA reports that in 2014 34% of regular video-game players were over 36. In 2016 the percentage of regular video-game players over 36 increased, to 39% [47, 48]. These patterns suggests that in the coming years video-game playing will become more common in older adults and finding non-video game players to run controlled experiments will become increasingly difficult. In the current study, findings from the participants that remained in the video game group strongly support the hypothesis that motivated older adults can benefit from video-game playing. The current findings are particularly important because in the future it will become increasingly difficult to find people interested in playing video-games that have not already done so on their own accord. It is likely to be even more difficult to find motivated non-video-game players in the future compared to non-musicians because learning to play video games is low cost, and can be done independently.

Future research should address how individual differences can contribute to the successful completion of 3D-platform training in older adults. The present results nonetheless represent a proof of concept that can support video game development that is more specifically tailored for older adults with the aim to achieve a higher rate of training compliance. The development of such a training tool could have a positive impact on healthy aging as decreased grey matter in the hippocampus is associated with increased risk for cognitive impairment and Alzheimer’s disease [1, 2], and decreased grey matter in the cerebellum is associated with decreased sensorimotor performance [37] and balance [38] in older adults.

This is the first study to show that video game training can have a positive effect on the hippocampal memory system of older adults. Further, we show that increased grey matter in the hippocampus is specific to 3D-platform video game training compared to an active control task that consists of music lessons. Future research should focus on the longevity of these observed effects, transfer to cognitive performance related to balance and spatial memory ability, and the development of a video game training tool tailored for older adults.

## Supporting information

S1 Fig Pre- and post-training scores for the MoCA **(a)** and short-term memory **(b)** tasks (*p < 0.05; +/- standard error).
(JPG)

S2 Fig Decreased grey matter in the **(a)** left hippocampus (x = -29, y = -18, z = -24; t = -6.34, p < 0.00005), **(b)** right hippocampus (x = 31, y = -7.9, z = -27; t = -6.25, p < 0.00005), **(c)** left cerebellum (x = -5, y = -64, z = -26; t = -8.81, p < 0.00005), **(d)** right cerebellum (x = 4, y = -67, z = -26; t = -8.10, p < 0.00005) and **(e)** right DLPCF (x = 34, y = 39, z = 9; t = -5.37, p < 0.0001) in the passive CON group was observed. Significant peaks of atrophy in hippocampus = HPC; significant peaks of atrophy in cerebellum = C; significant peak of atrophy in dorsolateral prefrontal cortex = DLPFC.
(PNG)

S3 Fig We also examined the relationship between training related changes in short-term memory performance and grey matter change at the significant peak in hippocampus revealed by the VID post-/pre-training contrast. This analysis included all participants from the three experimental groups. This produced a significant correlation where increased grey matter in the left hippocampus was related to increased short-term memory performance (*r*_(32)_ = 0.424, *p* < 0.05). Closed circles represent the CON group, open circles represent the MUS group and triangles represent the VID group.
(PNG)

S1 File Raw data values for behavioural analyses reported in this manuscript. (XLSX)

S2 File Sample MRI scan from dataset reported in this manuscript. (RAR)
