# Supplementary figures and images for: Playing Super Mario 64 increases hippocampal grey matter in older adults
Source: PLoS One. 2017 Dec 6;12(12):e0187779. doi: 10.1371/journal.pone.0187779 (PMC5718432; doi:10.1371/journal.pone.0187779)

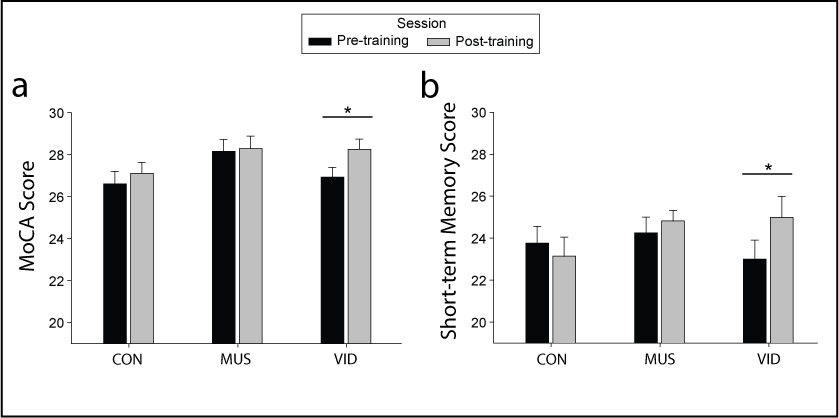

Supplement: S1 Fig — Pre- and post-training scores for the MoCA (a) and short-term memory (b) tasks (*p < 0.05; +/- standard error). (JPG) [file pone.0187779.s001.jpg]

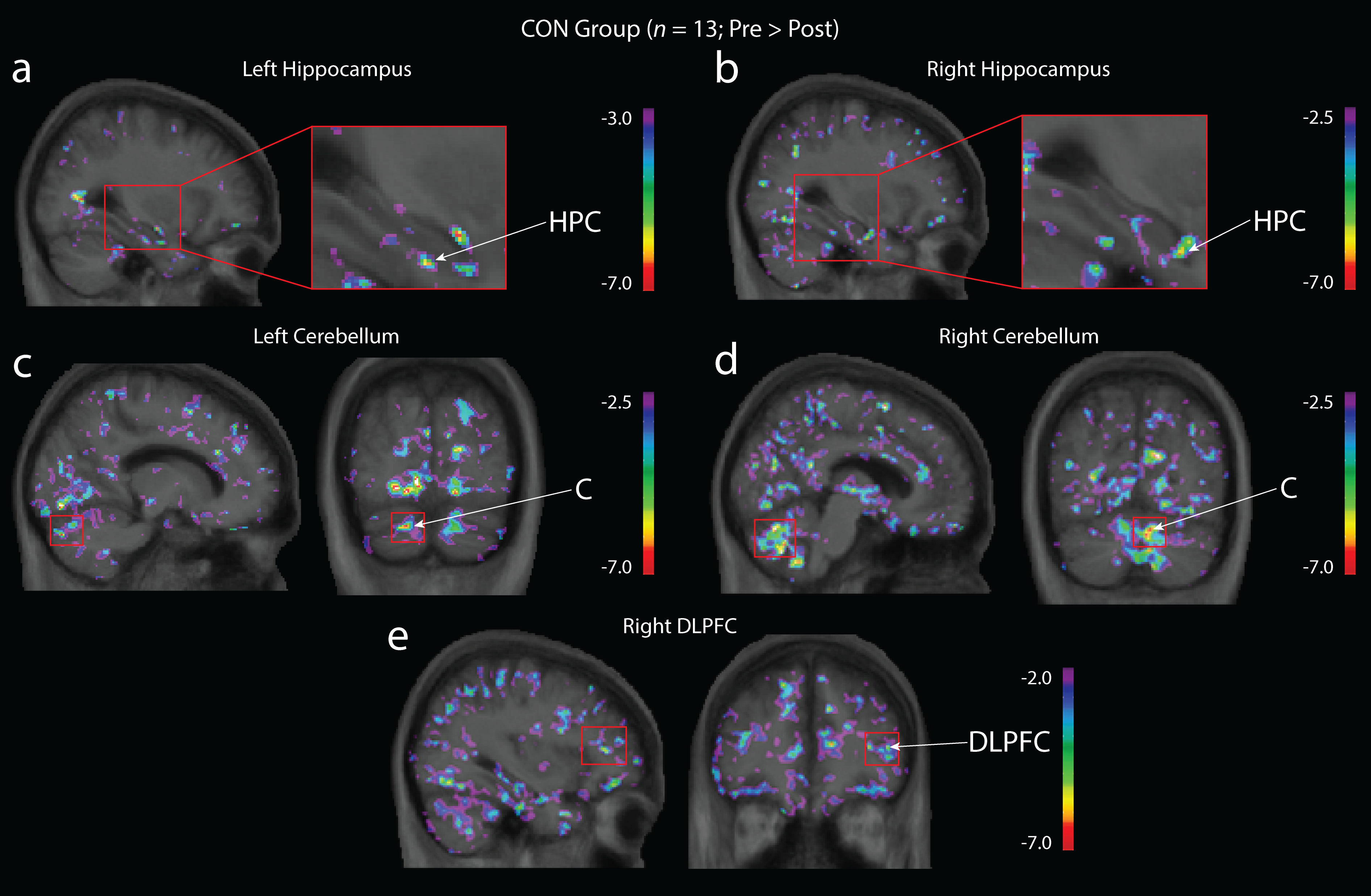

Supplement: S2 Fig — Decreased grey matter in the (a) left hippocampus (x = -29, y = -18, z = -24; t = -6.34, p < 0.00005), (b) right hippocampus (x = 31, y = -7.9, z = -27; t = -6.25, p < 0.00005), (c) left cerebellum (x = -5, y = -64, z = -26; t = -8.81, p < 0.00005), (d) right cerebellum (x = 4, y = -67, z = -26; t = -8.10, p < 0.00005) and (e) right DLPCF (x = 34, y = 39, z = 9; t = -5.37, p < 0.0001) in the passive CON group was observed. Significant peaks of atrophy in hippocampus = HPC; significant peaks of atrophy in cerebellum = C; significant peak of atrophy in dorsolateral prefrontal cortex = DLPFC. (PNG) [file pone.0187779.s002.png]

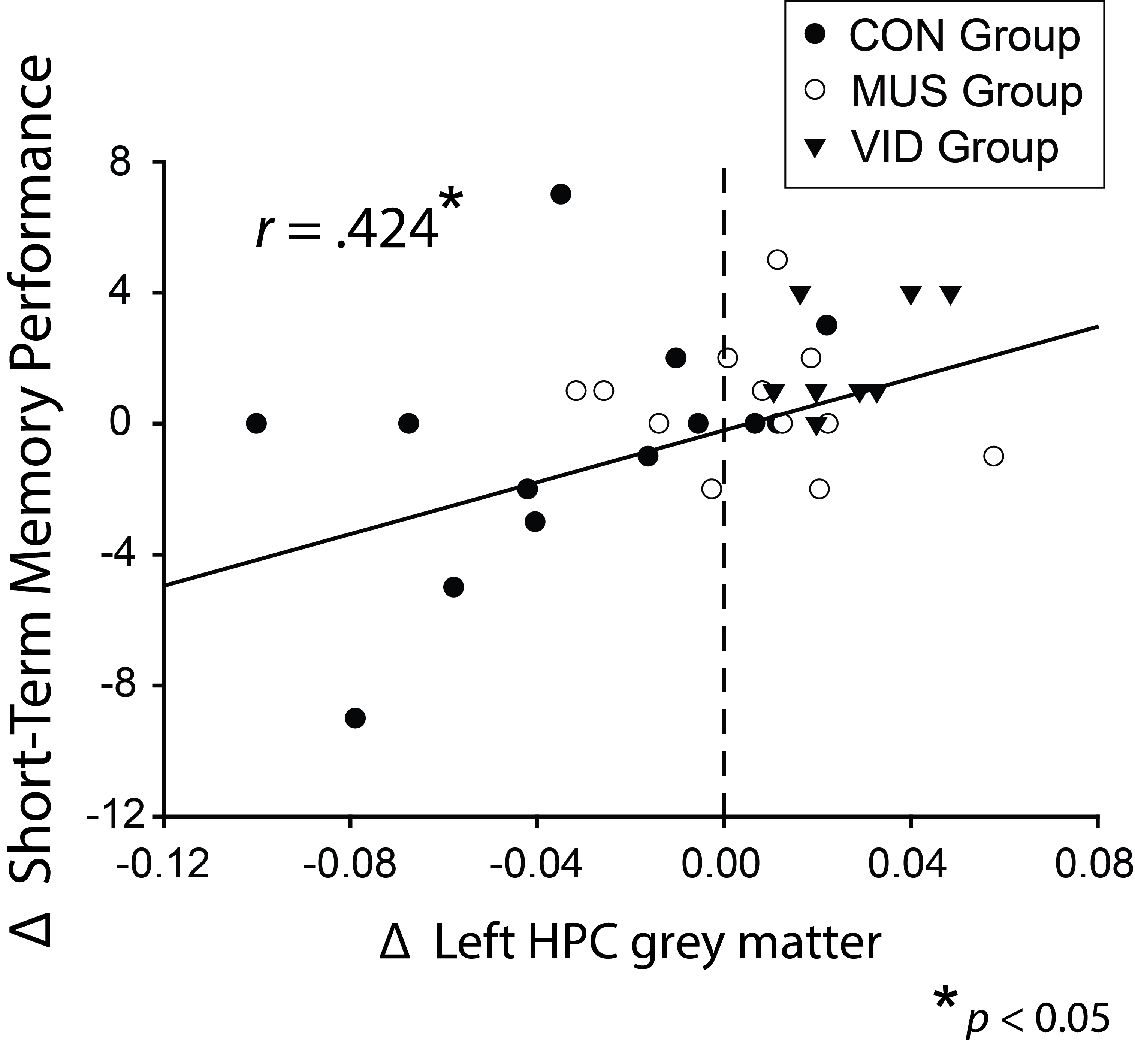

Supplement: S3 Fig — This analysis included all participants from the three experimental groups. This produced a significant correlation where increased grey matter in the left hippocampus was related to increased short-term memory performance (r(32) = 0.424, p < 0.05). Closed circles represent the CON group, open circles represent the MUS group and triangles represent the VID group. (PNG) [file pone.0187779.s003.png]
